# Testing a home-based model of care using misoprostol for prevention and treatment of postpartum hemorrhage: results from a randomized placebo-controlled trial conducted in Badakhshan province, Afghanistan

**DOI:** 10.1186/s12978-020-00933-8

**Published:** 2020-06-05

**Authors:** Dina F. Abbas, Shafiq Mirzazada, Jill Durocher, Shahfaqir Pamiri, Meagan E. Byrne, Beverly Winikoff

**Affiliations:** 1 Gynuity Health Projects, 220 East 42nd Street Suite 710, New York, NY 10017 USA; 2 Academic Projects Afghanistan, Aga Khan University, Co French Medical Institute for Children, Ali Abad, Kabul, Afghanistan; 3 Aga Khan Health Services Afghanistan (AKHS-A), An Agency of the Aga Khan Development Network (AKDN), Baghlan, Afghanistan

**Keywords:** Postpartum hemorrhage (PPH), Misoprostol, Treatment, Community health workers (CHWs), Prophylaxis, Home-births, Self-administration, Advance distribution

## Abstract

**Background:**

Postpartum hemorrhage (PPH) is the leading cause of maternal mortality worldwide. In Afghanistan, where most births take place at home without the assistance of a skilled birth attendant, there is a need for options to manage PPH in community-based settings. Misoprostol, a uterotonic that has been used as prophylaxis at the household level and has also been proven to be effective in treating PPH in hospital settings, is one possible option.

**Methods:**

A double-blind, randomized placebo-controlled trial was conducted in six districts in Badakhshan Province, Afghanistan to test the effectiveness and safety of administering 800mcg sublingual misoprostol to women after a home birth for treatment of excessive blood loss. Consenting women were enrolled prior to delivery and given 600mcg misoprostol to self-administer orally as prophylaxis. Community health workers (CHW) were trained to observe for signs of PPH after delivery and if PPH was diagnosed, administer the study medication (misoprostol or placebo) and immediately refer the woman. A hemoglobin (Hb) decline of 2 g/dL or greater, measured pre- and post-delivery, served as the primary outcome; side effects, additional interventions, and transfer rates were also analyzed.

**Results:**

Among the 1884 women who delivered at home, nearly all (98.7%) reported self-use of misoprostol for PPH prevention. A small fraction was diagnosed with PPH (4.4%, 82/1884) and was administered treatment. Hb outcomes, including the proportion of women with a Hb drop of 2 g/dL or greater, were similar between the study groups (misoprostol: 56.4% (22/39), placebo: 60.6% (20/33), *p* = 0.45). Significantly more women randomized to receive misoprostol experienced shivering (82.5% vs. placebo: 61.5%, *p* = 0.03). Other side effects were similar between study groups and none required treatment, including among the subset of 39 women, who received misoprostol for both of its PPH indications.

**Conclusions:**

While the study did not document a clinical benefit associated with misoprostol for treatment of PPH, study findings suggest that use of misoprostol for both prevention and treatment in the same birth as well as its use by lay level providers in home births does not result in any safety concerns.

**Trial registration:**

This trial was registered with ClinicalTrials.gov, number NCT01508429 Registered on December 1, 2011.

## Plain English summary

Medications, known as uterotonics, are used to prevent and treat excessive bleeding (postpartum hemorrhage, PPH) due to uterine atony, a potentially life-threatening complication that occurs when a woman’s uterus fails to contract after childbirth. The World Health Organization (WHO) recommends using misoprostol when oxytocin, the uterotonic of choice, is not an option. WHO’s recommendations also support use of misoprostol by lay health workers in home births to prevent the likelihood of PPH. Misoprostol has been proven to be effective in controlling PPH in large hospital-based studies; however, few studies exist that examine the role of misoprostol when administered by lay providers to treat PPH outside of health facilities. This study was conducted in rural Afghanistan to determine the effectiveness and safety of treating women delivering at home with misoprostol. All women were dispensed misoprostol (600mcg) in advance and counselled to swallow the pills immediately after delivery to prevent PPH. Community health workers (CHW) were present during the birth to observe for signs of PPH and to place four pills -- misoprostol (800mcg) or placebo -- under the tongue of women identified as bleeding heavily. The study did not find evidence for the effectiveness of misoprostol when comparing hemoglobin outcomes among women given misoprostol vs. placebo. Misoprostol was considered safe in the hands of CHWs, who were able to administer the medication correctly after identifying excessive bleeding. Women who received misoprostol to both prevent and treat PPH during the same birth did not report experiencing adverse effects or having safety concerns.

## Background

Misoprostol, an E-1 prostaglandin, is known for its efficacy in managing postpartum hemorrhage (PPH) [1–3]. Both international and country guidelines have incorporated misoprostol into clinical protocols and recommendations for prevention and treatment of PPH [4–6]. Misoprostol’s oral formulation and heat stable characteristic make it a versatile option to use at all levels of the health system, and evidence supports its use for both indications [7, 8]. While oxytocin is predominantly used in places where parenteral administration is possible, misoprostol remains a promising alternative, especially outside of health facilities. Indeed, one of the first studies conducted at the community level found misoprostol to be a promising therapeutic option for treating PPH after home deliveries in Tanzania when administered by traditional birth attendants [9]. However, its preventive use has gained more traction due to cumulating evidence and program experiences on misoprostol’s prophylactic use around the globe [7, 10]. Despite more than a decade of community research on the use of this medicine, there remains a gap in evidence on the use of misoprostol for both indications in the same delivery as part of a continuum of care, service delivery model at the community level.

Afghanistan was one of the first countries to pilot the advance distribution of misoprostol prophylaxis to women for self-use due to the high home delivery rate (66%) throughout the country [11]. The model leveraged the existing Basic Package of Health Services program designed to provide health services to rural populations and used community health workers (CHWs) to distribute misoprostol and educate women on its correct use [12]. CHWs, who staff health posts, are trained to do health promotion, offer basic health services, including antenatal and postnatal care, and identify obstetric complications. They also serve as the primary link between the community and health facilities, initiating referrals to seek additional care and facilitating the process [13].

Findings from the pilot study in Afghanistan demonstrated that the provision of misoprostol in advance to pregnant women by CHWs is safe and acceptable, and a feasible model to ensure access to misoprostol for PPH prevention [12]. These findings informed the Afghanistan Ministry of Public Health National PPH strategy on prevention of PPH in the community through advanced distribution of prophylaxis [14]. Under the current strategy, women who experience PPH after receiving misoprostol for prevention rely on transfer to a health facility for additional treatment. However, the logistical difficulties of transferring women in remote locations, such as rural Afghanistan, may hinder access to or delay care, leading to consideration of treatment options that are available to women at home.

Building on the national PPH strategy in Afghanistan, this study aimed to test the programmatic feasibility of equipping women with 600mcg oral misoprostol for self-use for PPH prevention after homebirth, in addition to training and supplying CHWs to offer a treatment dose of misoprostol (800mcg sublingually) when PPH is observed in the home. This study used a randomized, double-blind, placebo-controlled design to assess the effectiveness, safety, and acceptability of misoprostol use to treat PPH after its use prophylactically.

## Methods

The study was conducted in six districts (Zebak, Ishkashim, Wakhan, Shugnan, Nusai and Maimay) in Badakhshan Province in northeast Afghanistan. Existing CHW teams from 36 villages were selected in the six districts, along with three midwives who were recruited to supervise the CHWs. The principal investigator together with the country investigators organized an initial 4-day training on the study procedures with the local study coordinator and three midwives, responsible for monitoring, overall supervision, and data collection, followed by a 4-day training of CHWs. All trainings were conducted in the study districts; three separate trainings were held to train the CHWs in each district. Topics covered included supplies and logistics, as well as on how to counsel women on self-administering a prophylactic dose of misoprostol, diagnose PPH, administer the study medicine for PPH treatment, manage side effects, and refer women for additional treatment.

All pregnant women in the community were identified through the standard practice of community mapping carried out by CHWs for their catchment areas. As part of this mapping exercise, CHWs notate pregnant women by the household level. Using this information CHWs and study midwives visited pregnant women in their homes during the third trimester of pregnancy to enroll them in the study. To be eligible, a woman had to agree to have their pre- and post-delivery hemoglobin (Hb) measured, agree to have the CHW to be present in the room at the time of delivery to observe for signs of PPH, and participate in an exit interview, if diagnosed and treated for PPH. All willing women were taken through the informed consent process, after which the midwife measured pre- delivery Hb using a HemoCue Hemoglobin machine (Hemocue®, Angelholm, Sweden). In accordance with Afghanistan’s national PPH strategy [14], women received 600mcg misoprostol (3 tablets) during the third trimester visit and were counseled on how to self-administer the pills orally as PPH prophylaxis immediately after the birth of the baby (ies). Prophylaxis was packaged in boxes designed by the Ministry for Public Health for use in a pilot program that tested the advance distribution of misoprostol for the prevention of PPH [12]. Prior to the study launch, the protocol was approved by the Afghanistan Ministry of Public Health, Afghanistan Public Health Institute Institutional Review Board.

At the time of labor, women and their families used mobile phones or would send a messenger to notify the female CHW in the village so that she could be present for the delivery and monitor postpartum blood loss. CHWs were instructed to diagnose PPH in one of three ways: blood loss soaking through two cloths, via visual estimation, or visible deterioration in the woman’s clinical condition (e.g. profuse bleeding, paleness, faintness, rapid breathing). Using cloths to identify PPH is in line with guidance provided from the Ministry of Public Health Afghanistan on community-based management of PPH [14]. The study provided CHWs with cloths measuring one meter by one meter to use postpartum. Upon PPH diagnosis, the CHW administered the next sequential treatment packet of 800mcg misoprostol or matching placebo sublingually. The CHW concurrently referred the woman to seek care at a health facility. CHWs were provided mobile or satellite phones to help facilitate the referral process, which often entailed requesting the health facility to send an ambulance. If no ambulance was available, CHWs could request to use the study vehicle that had been provided by the study to assist with study monitoring and data collection.

CHWs documented the delivery characteristics for all participants, including overall condition, delivery complications, usage of misoprostol, and side effects. Data on the incidence of side effects, including fever was determined by observation or woman’s reporting. The study midwife conducted an in-person interview with all women who experienced PPH or received study treatment and took their post-delivery Hb three to five days after the delivery. For transferred cases, study midwives documented data on the care provided to participants with PPH at the health facility by interviewing the treating provider(s). Women who were referred for any complication prior to delivery became ineligible for the study and although their final outcomes were documented, the study did not record any data on interventions received at the facility.

The primary outcome was the proportion of women who experienced a Hb drop of 2 g/dL or greater pre- to post-delivery. Based on Hb outcomes from two large trials that studied the efficacy of misoprostol for PPH treatment in hospital settings [15, 16], we estimated that 75% of women in the misoprostol group would experience a Hb drop of 2 g/dL or greater. In the placebo group, we assumed that nearly all women (95%) would experience this important decline in Hb since no other immediate treatments are available to women in this area. Based on these assumptions, our calculated sample size established the need to enroll 70 treated cases with complete outcome data for analysis of the primary outcome (one sided test, power of 80%, α = 0.05). Additional clinical, secondary outcomes included mean post-delivery Hb, side effects, additional interventions, and rates of transfer for higher level care. Programmatic findings were also captured including acceptability of the treatment medicine, prophylaxis coverage, and CHW presence at the delivery.

All data were entered in Microsoft Access and then exported to SPSS 19.0 software (IBM, Chicago, IL, USA) for analysis. A modified intention-to-treat analysis was performed and included women who received the study treatment regardless of PPH diagnosis. Baseline characteristics were compared between study groups using Pearson χ^2^ or Fisher’s exact tests (as appropriate) for categorical variables and using independent t-tests for continuous variables. Log binomial regression was used to calculate relative risks and their associated 95% confidence intervals for categorical outcomes to assess the effect of misoprostol compared to placebo for the study’s main outcomes, including Hb decline ≥2 g/L, postpartum anemia, rates of transfer for higher level of care, and side effects. Statistical significance was defined as a *p*-value < 0.05.

### Randomization of treatment

The randomization scheme was computer-generated by Gynuity Health Projects in New York and not revealed until after all study participants had been discharged from the study, data collection was completed, and the database was cleaned. The randomization code was created in blocks of four with a one-to-one allocation ratio between misoprostol and placebo. Due to logistical constraints related to the supply of the drug, three independent randomization codes were created for each study midwife (3). Treatment packets consisted of four tablets (200mcg) of misoprostol (GYMISO, Linepharma, Paris, France) or matching placebo (Linepharma, Paris, France). Each packet was consecutively numbered to maintain blinding and color coded to help the CHWs adhere to the randomization sequence. CHWs were supplied with four treatment packets at a time to use for cases diagnosed with PPH. Regular monitoring visits by study midwives and investigators were conducted with CHWs to ensure that the packets were used per study procedures. Participants, study investigators and staff, including the CHWs, were blinded to the allocation code.

## Results

**Fig. 1:**
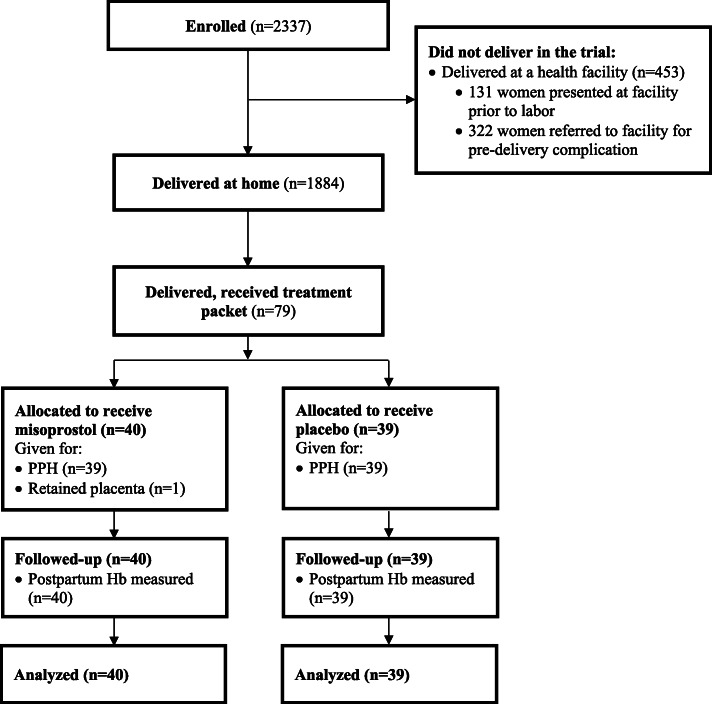
Consort flow diagram: trial profile

The study enrolled 2337 women, of whom 1884 delivered at home and 453 delivered at a health facility (Fig. 1). Enrollment commenced on 11 August 2012 and the study ended on 28 February 2016 when the last enrolled woman delivered. Of the 2206 women who began the labor process at home, the CHW arrived at the home before or during the delivery in 1871 (82.4%) cases. The majority of women (1860/1884, 98.7%) who delivered at home took the misoprostol prophylaxis without any difficulties. PPH was diagnosed among 4.4% of home births (82/1884). In 1540 (81.7%) deliveries, the CHW used the cloths provided by the study to help monitor postpartum blood loss, which was the primary tool used to diagnose PPH in 91.5% (75/82) of cases. In the remaining cases, CHWs relied on visual estimation and the woman’s condition to assess whether the postpartum bleeding was more than normal.

Four women diagnosed with PPH did not receive study treatment. In one case the woman experienced a lock jaw and could not receive treatment, one woman refused because of having experienced severe shivering after administration of prophylaxis, in one case the CHW was not present at the home so the woman was transferred to the facility, and in one case a skilled midwife was present at the home and administered oxytocin.

Study treatment was administered to 79 women (misoprostol: 40, placebo: 39), who are included in the primary outcome analysis (see Fig. 1). The CHW was not present for the birth in 12 (15.2%) cases where study drug was administered (misoprostol: 4 placebo: 7, *p* = 0.35). In these cases a family member contacted the CHW and requested the CHW to come to the home to treat the woman after the family or woman herself had identified a complication, which in 11 cases was PPH and in one case was retained placenta. Participant baseline characteristics were similar between the two groups, except for the duration of iron folate intake prior to delivery, which was longer in the placebo group compared to the misoprostol group (Table 1). Among the group of women diagnosed and treated for PPH, nearly all had reported taking 600mcg oral misoprostol after delivery to prevent the occurrence of PPH (misoprostol: 97.5% (39/40); placebo: 92.3% (36/39), *p* = 0.296).

**Table 1:** Background characteristics by study group*

	Misoprostol (800mcg SL) (***n*** = 40)	Placebo (***n*** = 39)
Age, mean ± SD	24.7 ± 4.8	26.0 ± 5.5
Parity, mean ± SD	3.1 ± 1.9	2.9 ± 1.8
Woman categorized as literate	8 (20.0)	8 (20.5)
Iron folate tablets taken during pregnancy	38 (95.0)	38 (100) ^a^
Duration during which iron folate tablets were taken (months), mean + SD	1.9 ± 1.5	2.4 ± 2.3 ^a^
Pre-delivery Hb (g/dL) ^b^		
mean ± SD	12.4 ± 1.4	12.1 ± 1.8
(range)	(9.0–15.9)	(7.7–15.8)

Abbreviations: *Hb* hemoglobin, *SL* sublingual

* Data presented as n (%) unless otherwise stated

^a^*n* = 38; data on iron folate missing for one case in the placebo arm

^b^ In six cases (misoprostol, *n* = 1; placebo, *n* = 5) pre-delivery Hb was not measured due to enrollment in the study at the time of the delivery

Complete Hb data including pre- and post-delivery was documented for 72 women who received the study treatment (see Table 2). The proportion of women who experienced a pre- to post-delivery Hb drop of 2 g/dL or greater (the study’s primary outcome) was not significantly different between the study groups (misoprostol: 22 (56.4%), placebo: 20 (60.6%), *p* = 0.45). Rates of postpartum anemia, defined as Hb ≤ 8 g/dL in this setting, were also similar (Table 2).

**Table 2:** PPH outcomes by study group*

	Misoprostol (800mcg SL) (***n*** = 40)	Placebo (***n*** = 39)	RR 95% CI	***P*** value
**Primary outcome**				
Hb drop ≥2 g/dL ^a^	22 (56.4)	20 (60.6)	0.93 (0.61, 1.45)	0.45
**Secondary outcomes**				
Post-delivery Hb (g/dL), mean ± SD ^b^	10 ± 1.7	9.9 ± 1.7		0.94
Postpartum Hb ≤ 8 g/dL ^b^	7 (17.5)	8 (21.1)	0.83 (0.29, 2.33)	0.78
Pre to post-delivery Hb drop (g/dL), mean ± SD ^a^	2.4 ± 1.5	2.4 ± 1.8		0.99
Hb drop ≥3 g/dL ^a^	13 (33.3)	12 (36.4)	0.92 (0.45, 1.89)	0.81
Transferred to facility from home	18 (45.0)	15 (38.5)	1.17 (0.66, 2.11)	0.56
Administered IV oxytocin at facility	17 (42.5)	14 (35.9)	1.18 (0.64, 2.21)	0.55
Other interventions (Ns listed):				
Administered ergometrine	2	6		
Manual removal of placenta	2	1		
Suturing/tear repair	1	1		
Hysterectomy/other surgery	0	0		

Abbreviations: *Hb* hemoglobin, *SL* sublingual, *RR* relative risk, *CI* confidence interval

^*^ Data presented as n (%) unless otherwise stated

^a^ Pre and post Hb measures available for *n* = 39 in the misoprostol arm and *n* = 33 in the placebo arm

^b^ In one case (placebo) no post-delivery Hb was available for the one maternal death

All women who received study treatment were referred by the CHW to a health facility for further evaluation per study protocol and national guidelines recommending referral when complications occur in the home. Transfers occurred for fewer than half of women who were diagnosed with PPH and received study treatment and was not statistically different between study groups (Table 2). The majority of women who were successfully transferred to a facility were offered additional uterotonics (93.9%). IV oxytocin was primarily used in the facilities, and the proportion of women who received this uterotonic was similar between study groups. Provision of additional care aside from uterotonics was rare and included suturing, bimanual compression, and manual removal of placenta (Table 2). All women transferred due to PPH were discharged from the facilities in stable condition.

There was one maternal death in the study. The CHW was not present for the delivery and was contacted after the birth by the family over concerns about postpartum bleeding. The CHW administered a treatment packet (placebo) and advised the family to transfer the woman to a health facility. The family, who felt the woman’s condition was improving, took into consideration the heavy snowfall and non-existent road to the health facility, and decided to wait till dawn to transfer the woman, who died overnight. There were no other serious events in the study, including hysterectomies, blood transfusions or surgeries among women enrolled.

The proportion of women randomized to the misoprostol group who experienced shivering was higher than for women in the placebo group (33 (82.5%) vs. 24 (61.5%), *p* = 0.05) (Table 3). More women in the placebo arm reported that they experienced fever; this difference was not significantly significant (7 (17.9%) vs. 4 (10%), *p* = 0.35). Rates of nausea, vomiting and fainting were similar between study groups. Of the 40 women who were randomized to receive 800 mcg misoprostol sublingually as treatment, 39 (97.5%) also took 600 mcg oral misoprostol prophylaxis during the third stage of labor. This subset of women, who had used 600mcg oral misoprostol for prophylaxis and then received 800mcg sublingual misoprostol for treatment, did not experience any adverse effects or side effects that required treatment. Among women randomized to the study groups, overall acceptability of side effects was high (misoprostol: 31/35 (88.6%), placebo: (34/35) 97.1%; *p* = 0.36) (Table 3). None of the women who received study treatment reported any challenges with breastfeeding. There were no reports of incorrect administration of study treatment packets.

**Table 3:** Side effects and adverse events by study group*^a^

	Misoprostol (800mcg SL) ***n*** = 40	Placebo ***n*** = 39	RR 95% CI	***P*** value
Shivering	33 (82.5)	24 (61.5)	1.34 (0.98, 1.74)	0.05
Fever	4 (10.0)	7 (17.9)	0.56 (0.14, 1.97)	0.35
Vomiting	6 (15.0)	7 (17.9)	0.84 (0.27, 2.58)	0.72
Fainting	2 (5.0)	1 (2.6)	1.95 (0.14, 54.1)	1.0
Nausea	6 (15.0)	5 (12.8)	1.17 (0.34, 4.19)	0.78
Side effects reported as acceptable ^b^	31/35 (88.6)	34/35 (97.1)	0.91 (0.86, 1.06)	0.36
Maternal death	0	1		
Neonatal death	0	1		

Abbreviations: *SL* sublingual, *RR* relative risk, *CI* confidence interval

* Data are presented as n (%)

^a^ Among those who received the study treatment, all received 600mcg oral misoprostol as prophylaxis with the exception of 4 women (1 in misoprostol arm; 3 in placebo arm)

^b ^In nine cases participants did not report on acceptability of side effects (5 in misoprostol arm; 4 in placebo arm)

## Discussion

While findings showed that this home-based model of care using misoprostol to manage PPH is programmatically feasible and safe in this setting, treatment with misoprostol, compared to placebo, did not significantly reduce the proportion of women who experienced Hb drop ≥2 g/dL, which served as a proxy for measuring the effectiveness of the intervention in this study. Over half of women randomized to receive treatment with misoprostol experienced a decline in Hb ≥2 g/dL (56%), compared to 61% in the placebo arm. Irrespective of the receipt of misoprostol or placebo for treatment of PPH after home delivery, the rate of transfer was also similar. With the exception of one maternal death in this study, all women who received either placebo or the active drug were reported to be in stable condition at their exit interview 3–5 days postpartum, including women who did not receive higher level of care.

Similar findings were reported in a concurrent randomized controlled trial conducted in Pakistan that tested the same misoprostol regimens for prevention and treatment of PPH among home births attended by traditional birth attendants in rural Pakistan [17]. The primary outcome analysis in that study also found that the proportion of women who had a decline in Hb drop ≥2 g/dL was lower in the misoprostol treatment arm (20/43, 47%), compared to placebo (19/33, 58%), although this finding was not statistically significant (*p* = 0.335). Likewise, a combined analysis of the Pakistan results [17] together with the Afghanistan findings do not confirm any association between treatment with misoprostol for PPH and the incidence of Hb drop ≥2 g/dL (Relative Risk 0.86 95% CI 0.6–1.2). In both studies, severe outcomes from PPH were rare and none of the cases treated with the study medication required surgical care or blood transfusion. There were two maternal deaths from PPH (1 in the Afghanistan study, 1 in the Pakistan study) – both occurred among women not treated with misoprostol and who did not receive higher level care as a result of decisions made by family members that impeded immediate transfer. It is evident from these maternal deaths that transfer for higher level care is sometimes influenced by factors other than the clinical condition of the woman, which make it an unreliable solution for managing PPH [17]. In addition to making treatment options for PPH available at the community level, it would therefore be prudent to develop and test additional community-based approaches that include robust educational components about the dangers of PPH and the importance of seeking care.

In the Afghanistan study, of note, is the striking difference in transfer rates among women who experienced complications before or at the time of delivery, which was 87.2% (355/407), as compared to women transferred due to PPH (41.7%, 33/79). Among the PPH cases, it is plausible that if bleeding decreased or was controlled, the urgency to seek care may have subsided over time. However, a surprising finding is the null result with regard to care-seeking behaviors for women diagnosed with PPH and randomized to treatment with misoprostol or placebo, especially in light of earlier findings from community-based research from Tanzania [9]. That study had documented a low referral rate for PPH (2%, 8/454) when equipping TBAs with misoprostol to administer for treatment of PPH in home deliveries, compared to a referral rate of 19% (76/395) in the non-intervention (control) period [9]. An important difference between the Tanzania model and the one implemented in Afghanistan, however, is the provision of misoprostol directly to women to use as prophylaxis. In our study, nearly all women (1860/1884, 98.7%) who delivered at home took the misoprostol to prevent heavy bleeding. The effect of the prophylactic dose may have contributed to the favorable outcomes in our study and played a role in bleeding cessation even among women for whom PPH was diagnosed.

The findings from this study and others [9, 17, 18] also contribute to the evidence base on the safety of offering misoprostol for PPH treatment in community settings. There were no safety concerns or severe side effects reported among women who received misoprostol for treatment in addition to prophylaxis in this study or the Pakistan study [17]. However, implementing strategies to ensure both prophylaxis and treatment regimens of misoprostol are available to the same woman, in case hemorrhage occurs, may place an undue logistical and resource burden on programs. Another community-based approach that offers 800mcg sublingual misoprostol as a preemptive treatment, i.e. “secondary prevention” to women with above-normal blood loss is a recommended alternative to a universal prophylaxis with 600mcg oral misoprostol and may help overcome potential systems-related challenges including supply management, sustainability and costs [4, 18].

A key strength of this study is the rigorous data collection that took place over a three- and half-year time period documenting the use of misoprostol for prevention and treatment of PPH as well as community-based practices and the CHW’s role in obstetric care in remote settings. Despite WHO’s recommendations in support of misoprostol for PPH treatment in situations when oxytocin is not available [5], community-based programs have typically shied away from introducing misoprostol to treat PPH citing concerns about the complexity of training lay providers or women to identify and subsequently treat PPH. Instead programs and evaluations have focused on use of misoprostol as prophylaxis in home births either through self-use or administration by a lay provider [8, 10]. Our study findings support existing evidence that CHWs can identify complications, including PPH, make referrals to seek care, and facilitate transfers for higher level care [19, 20], as well as provide misoprostol as a treatment option. The study also built on previous community-based strategies implemented in Afghanistan and elsewhere that provided women with 600mcg oral misoprostol to self-administer after childbirth for prevention of PPH [12, 21]. The findings from our study confirmed that women can safely keep and correctly use the misoprostol they received in advance during their third trimester as prophylaxis. These data are valuable when considering the design of community-based models to deliver maternal care in settings where there is a shortage of skilled providers.

This study does have some limitations. First, the study was underpowered to detect differences due to the lower than expected incidence in the primary outcome (Hb drop ≥2 g/dL). Also, of consideration, the study provided all CHWs with a monetary incentive to be present at the birth. Moreover, CHWs received phones or phone credit and study vehicles were available to help facilitate transfer for any complication. These additional resources may have improved communication and contributed to the programmatic success of this service model; thus, limiting the generalizability of our findings. For instance, access to the study vehicle might have facilitated the transfer of women with complications identified by the CHW to a facility prior to delivery, thereby reducing the number of women with potential comorbidities from the pool of home births enrolled in our study, which likely contributed to better outcomes for women. Indeed, the presence of other comorbidities is known to increase the risk of developing PPH and its sequelae, including death [22]. In the present study, one death occurred out of > 2000 deliveries, and in other remote settings communities, without an injection of additional resources, similar outcomes may not be achievable.

## Conclusions

Misoprostol is currently the only pill-based uterotonic option that could be offered at the community level to treat PPH and is supported in international recommendations on PPH management as a viable alternative to oxytocin [4, 5]. While the study did not show a benefit of misoprostol treatment on Hb outcomes, the provision of sublingual misoprostol (800mcg) as an intermediary measure to women in remote settings has been shown to be safe, including among women given the same medicine for prophylaxis (600mcg oral misoprostol). Furthermore, it is more probable than not, that misoprostol helps control bleeding when caused by uterine atony, based on what is known about the clinical effect of misoprostol from larger hospital trials [15, 16]. Possibly due to the small sample in this study and other aspects of the study design, it was not possible to document a clinical advantage to offering misoprostol compared to placebo in this study. Nevertheless, programmatic lessons from this study that integrated two models of care offering misoprostol as prophylaxis and as treatment for PPH affirm that the management of this condition requires a pathway of care that empowers women, families and community members for achieving timely care. In addition to incorporating pills, community models need to consider awareness campaigns to stress the importance of births at facilities or transfer, as well as additional resources that may facilitate earlier identification of complications and transfer.

## Data Availability

The dataset generated and analyzed during the current study are available from the corresponding author on reasonable request.

## Abbreviations

CHW: Community health workers
Hb: Hemoglobin
PPH: Postpartum hemorrhage
WHO: World Health Organization
